# Gut Microbiota Resilience: Definition, Link to Health and Strategies for Intervention

**DOI:** 10.3389/fmicb.2020.572921

**Published:** 2020-09-15

**Authors:** Shaillay Kumar Dogra, Joel Doré, Sami Damak

**Affiliations:** ^1^Nestlé Research, Société des Produits Nestlé SA, Lausanne, Switzerland; ^2^Université Paris-Saclay, Institut national de recherche pour l’agriculture, l’alimentation et l’environnement, MetaGenoPolis, AgroParisTech, Microbiologie de l’Alimentation au Service de la Santé, Jouy-en-Josas, France

**Keywords:** microbiome, resilience, probiotic, fiber, health

## Abstract

The gut microbiota is a new frontier in health and disease. Not only many diseases are associated with perturbed microbiota, but an increasing number of studies point to a cause-effect relationship. Defining a healthy microbiota is not possible at the current state of our knowledge mostly because of high interindividual variability. A resilient microbiota could be used as surrogate for healthy microbiota. In addition, the gut microbiota is an “organ” with frontline exposure to environmental changes and insults. During the lifetime of an individual, it is exposed to challenges such as unhealthy diet, medications and infections. Impaired ability to bounce back to the pre-challenge baseline may lead to dysbiosis. It is therefore legitimate to postulate that maintaining a resilient microbiota may be important for health. Here we review the concept of resilience, what is known about the characteristics of a resilient microbiota, and how to assess microbiota resilience experimentally using a model of high fat diet challenge in humans. Interventions to maintain microbiota resilience can be guided by the knowledge of what microbial species or functions are perturbed by challenges, and designed to replace diminished species with probiotics, when available, or boost them with prebiotics. Fibers with multiple structures and composition can also be used to increase microbiota diversity, a characteristic of the microbiota that may be associated with resilience. We finally discuss some open questions and knowledge gaps.

## Introduction

The human gut harbors trillions of microbes, essentially bacteria, counting more than 3000 different bacterial species with each individual harboring 200 to 300 species (Qin et al., 2010). This microbial ecosystem, the microbiota, plays a crucial role in human physiology and health (Flint et al., 2012; Gentile and Weir, 2018). Research on gut microbiota has historically focused on associations with diseases such as Irritable Bowel Syndrome (Menees and Chey, 2018), Inflammatory Bowel Disease (Ott et al., 2004; Manichanh et al., 2006), allergy (Ho and Bunyavanich, 2018), diabetes (Sharma and Tripathi, 2018), cancer (Baba et al., 2017; Dahmus et al., 2018), asthma (Fujimura and Lynch, 2015), and obesity (Furet et al., 2010). A few studies have gone beyond association and suggested causative effect of the microbiota on certain diseases, based either on fecal transplants in humans (Vrieze et al., 2012; Philips et al., 2017; Palleja et al., 2018; Chen et al., 2019; Costello et al., 2019; Kang et al., 2019) or on human microbiota transfer to germ-free mice (Le Roy et al., 2013; Duca et al., 2014; Peng et al., 2014; Kelly et al., 2016; Llopis et al., 2016; Schaubeck et al., 2016; Routy et al., 2018; Sharon et al., 2019; Kim et al., 2020). The adult gut microbiota is fairly stable while constantly being influenced by the host and by multiple external factors. Triggered by particularly strong stressors the gut microbiota may be critically modified and this could impact individual’s health.

## High Variation of Microbiota Composition and How to Define a Healthy Microbiota

Defining a healthy microbiota is very important for being able to prevent or correct dysbiosis and minimize its impact on health. However, the composition of the microbiota is very diverse and highly variable, depending on diet (Orwin and Wardle, 2004; Wu et al., 2011; Johnson et al., 2019), geographic location (Gupta et al., 2017; He et al., 2018), ethnicity (Deschasaux et al., 2018), level of exercise (O’Sullivan et al., 2015; Cook et al., 2016; Mach and Fuster-Botella, 2017; Barton et al., 2018), medication use including but not limited to antibiotics (Zaura et al., 2015; Imhann et al., 2016; Jackson et al., 2018), and genetics (Goodrich et al., 2014). In addition to these intrinsic and extrinsic modulators, a large part of the microbiota variation between individuals cannot be explained by any specific factor (Walker et al., 2011; Wu et al., 2011).

The high degree of variability makes it difficult to define a normal or healthy microbiota (Mardinoglu et al., 2018; McBurney et al., 2019). Yet some parameters such as increased diversity, gene richness, or proportion of butyrate producers are often considered as features of a healthy gut microbiota (reviewed in Lemon et al., 2012; Lloyd-Price et al., 2016). The impact on host parameters such as the gut barrier function (Natividad and Verdu, 2013) and immunity (Ye et al., 2018) can also be taken into consideration when determining if the gut microbiota is healthy or dysbiotic.

Alternatively, in case of gut microbial ecosystems, resilience can be used as a surrogate marker of a healthy ecosystem (Allison and Martiny, 2008; Banning and Murphy, 2008; Lozupone et al., 2012; Greenhalgh et al., 2016; Sommer et al., 2017; Fraccascia et al., 2018). Resilience is the property of an ecosystem to resist changes under stress or to quickly and fully recover from the perturbations (Ingrisch and Bahn, 2018, Box 1).

## Microbiota Resilience

**FIGURE 1:**
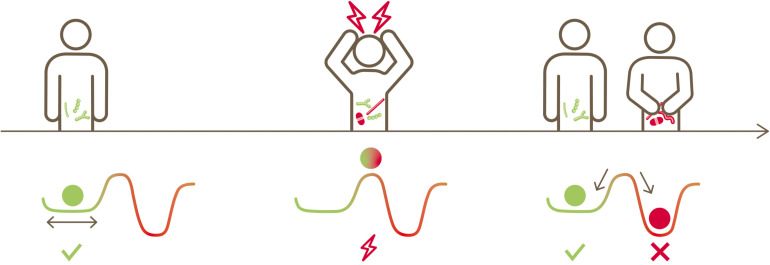
Conceptual illustration depicting the native state of an ecosystem, its perturbation, the possibility of returning to its original state or transitioning to a new state (alternative stable state) (inspired by Folke et al., 2004).

Linked together with functional and life-sustaining inter-dependencies, bacteria inhabiting the human gut are structured as a complex ecosystem with multiple cross-talk (Costello et al., 2012; Coyte et al., 2015; Gilbert and Lynch, 2019). Such a gut microbial ecosystem evolves and establishes itself during early life from neonate to infants to toddlers (Backhed et al., 2015; Dogra et al., 2015; Stewart et al., 2018) and stays mostly stable in adult life (Mehta et al., 2018). External stress factors such as extreme dietary changes (David et al., 2014; Mardinoglu et al., 2018), infections (Hsiao et al., 2014), antibiotics usage (Jernberg et al., 2007; Dethlefsen and Relman, 2011; Willing et al., 2011; Palleja et al., 2018) or other medications, including members of all therapeutic classes (Le Bastard et al., 2018; Maier et al., 2018) perturb this ecosystem. Subsequently, this gut microbial ecosystem may or may not return to its original state. This is conceptually illustrated in Figure 1, inspired by Folke et al. (2004). A resilient microbiota will return to its original state of equilibrium after being subjected to a perturbation, whereas a non-resilient microbiota will shift to an altered new state.

**Box 1. Definitions**

Baseline is the state of an ecosystem before perturbation.

Impact is the change in the ecosystem because of a stressor.

**FIGURE 2:**
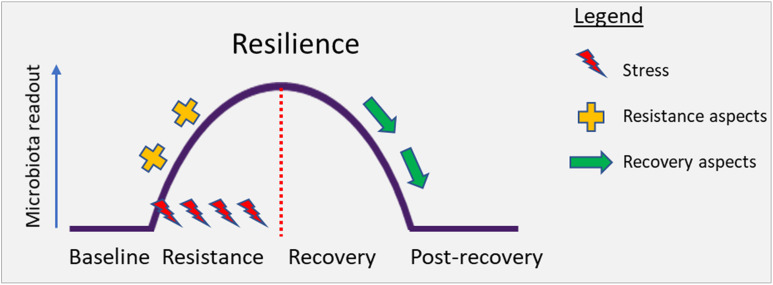
Conceptual illustration depicting the perturbation of the gut microbiota under stress. Resilience describes the capacity of an ecosystem to maintain its state or recover from disturbances. Resilience is determined by the capacity to reduce the impact (resistance) and to recover from the impact of disturbance (recovery) (Ingrisch and Bahn, 2018).

Resilience is the property of an ecosystem to maintain its state and recover from perturbations. The capacity of the system to persist during the impact (resistance) and to return to baseline after the impact of disturbance (recovery) determines overall resilience. Conceptually, this has been illustrated in Figure 2 below.

It can be argued that the microbiota of a healthy individual with the ability to return quickly and fully to baseline after a challenge is a healthy microbiota because this resilience may prevent the establishment of a new equilibrium and drift toward dysbiosis, with negative impact on the individual’s health (Sommer et al., 2017).

The concept of gut microbiota resilience has been discussed in several reviews and perspective articles (Relman, 2012; Lloyd-Price et al., 2016; Sommer et al., 2017). As recently proposed, a model combining challenge tests and biomarkers that can inform on the dynamics of microbiota recovery allows to document resilience as an indicator of health (Taroncher-Oldenburg et al., 2018). However, these proposals remain theoretical and to our knowledge, experimental validation of models for quantification of resilience has not been reported.

## What Are the Characteristics of a Resilient Microbiota?

Diversity may be a positive contributor to resilience. Tap et al. (2015) found that the human gut microbiota richness increases its stability when challenged by increased dietary fiber intake. Another human study showed that a weaker antibiotic-induced perturbation of the microbiota is linked to higher pre-challenge microbiota diversity (Raymond et al., 2016).

Host immune status may also affect microbiota resilience. Two studies have shown that genetic ablation of the bacterial sensor nod 2 in mice leads to impaired recovery of the microbiota from antibiotic perturbation (Goethel et al., 2019; Anderson et al., 2020). However, a third study using aroA-deficient *Salmonella* as challenge did not find any effect of the nod proteins on microbiota resilience (Robertson et al., 2016). Nod is an intra-cellular sensor of immune signals especially sensitive to specific peptidoglycan structures, hence reactivity may depend on the pathogen used in the challenge. These elegant basic research studies suggest that stability of host-microbiota symbiosis may be another important metric to assess resilience.

A few authors have attempted to model stability and recovery of the gut microbial ecosystem. Shaw et al. (2019) used stability landscape frameworks to model recovery of the gut microbiota after treatment with antibiotics. Certain other methods, such as increased auto-correlation and variance of the variables in the ‘dominant’ groups, can be early indicators of critical transitions to alternative stable states (Scheffer et al., 2009; Liu et al., 2015). One study focused on defining the ‘tipping elements’ in the microbiota by looking into the distribution of specific microbiota species and found bi-modal distributions that associate with host parameters. Some of the bacteria were either hardly present or quite abundant, depending on certain host factors, and may be those associated with the shift to an altered state (Lahti et al., 2014).

## Assessing Microbiota Resilience Experimentally

There are a few formulae for resilience described in the literature (Orwin and Wardle, 2004; Banning and Murphy, 2008; Fraccascia et al., 2018; Lourenco et al., 2018; Ye et al., 2018). Most of these were reviewed recently and categorized into a conceptual framework (Bahn and Ingrisch, 2018; Ingrisch and Bahn, 2018; Yeung and Richardson, 2018). Briefly, these formulae can be classified into three types of metrics: one describes change in relation to the baseline from the pre-disturbance state, thereby assessing to which extent the system gets perturbed; the other two metrics look into recovery after disturbance. This recovery can either be formulated as relative to baseline or relative to magnitude of perturbation. The former emphasizes how well the system has returned to its pre-perturbation state. The latter emphasizes how much it has recovered from the impact of perturbation.

In order to assess microbiota resilience experimentally, one must apply a challenge to the microbiota and measure some or all the above-mentioned parameters. A few studies have used an antibiotic or dietary challenge to perturb the microbiota and assess the impact of various interventions on this perturbation (Engelbrektson et al., 2009; Odamaki et al., 2016; Rodriguez-Morato et al., 2018).

The David et al. (2014) study provides a good model to study microbiota resilience in human trials. The challenge it used is diet of animal origin (e.g., meat, cheese), made of 70% fat, 0% carbohydrates, and 30% protein. Microbiota composition changed significantly as early as after 2 days of dietary intervention and returned to baseline 6 days after the end of the diet challenge. The advantage of using a dietary challenge is that it can be standardized, and does not raise ethical issues, as using antibiotics in healthy subjects might do.

A resilience index can be computed by using parameters that are known to be modified by stress such as microbiota composition, function and/or metabolites. The resilience index can be used to give a quantitative measure of how much the microbiota has deviated and how quickly and fully it has recovered, thereby quantifying resilience. Such a resilience index provides a measure which can possibly be used to compare different stressors across studies.

Ideally, one should be able to assess the microbiota resilience of an individual without applying a stressor. Machine learning with comprehensive and big data may help design algorithms based on microbiota and host parameters that can be used to predict microbiota resilience. One such parameter is α-diversity. This is supported by a human study showing that a weaker antibiotic-induced perturbation of the microbiota is linked to higher pre-challenge microbiota α-diversity (Raymond et al., 2016). A study using microbiota transfer into germ-free mice showed that the magnitude of antibiotic induced perturbation was donor dependent (Lavelle et al., 2019), giving ground to the hypothesis that it could be possible to predict resilience by analyzing the baseline microbiota in the absence of a challenge.

## What Is the Relationship of Gut Microbiota Resilience to Health?

As discussed above, throughout the lifetime the gut microbiota is subjected to repeated and diverse challenges, including unhealthy diet, medications, alcohol, intense exercise, and pathogens, to just name some. Decreased ability to resist these challenges or to return quickly and fully to the pre-challenge state may lead to a new equilibrium and dysbiosis, which may contribute to the development of chronic non-communicable diseases (CNCDs). The diminished ability of the microbiota to return to baseline and establishment of a new equilibrium has been observed in human subjects following treatment with antibiotics (Dethlefsen and Relman, 2011). Thus, intervening to maintain the microbiota in a resilient state may constitute a measure to delay or prevent the development of microbiota related CNCDs. It is noteworthy that the microbiota constitutes an easily accessible “organ” for intervention, although how to stably modulate the microbiota may be less evident.

This theory of a link between microbiota resilience and health is attractive but there are very few data to support it. In a human study, Mondot et al. (2016) defined microbiota robustness as high species richness and high inter-OTU (operational taxonomic unit) correlation. They found that microbiota robustness was positively associated with Crohn disease remission after ileocolic resection (Mondot et al., 2016).

## What Are the Possible Means of Intervention?

### Fibers

Knowing the characteristics of a resilient microbiota will help design the interventions aimed at increasing resilience. While this knowledge is currently incomplete, it is already known that diversity may be a contributing parameter (Tap et al., 2015; Raymond et al., 2016) and dietary fibers may be a way of increasing microbiota diversity. Dietary fibers are carbohydrate polymers that are not digested nor absorbed in the upper gastrointestinal tract and reach the colon where they are subjected to bacterial fermentation. Many studies have shown that fibers impact the composition and function of the microbiota, especially the production of short chain fatty acids (for review see, Holscher, 2017). Studies of humans in different geographical locations showed that greater dietary fiber intake is associated with increased gut microbiota diversity (Rampelli et al., 2015) and human intervention studies have revealed that dietary fiber and whole grain intake increase the diversity of the gut microbiota (Martinez et al., 2013; Tap et al., 2015). Microbiota diversity could in principle also be increased by a complex mix of dietary fibers providing a wide range of structures and monosaccharide units as indicated by at least one mouse study (Cheng et al., 2017). A study in mice has shown that fibers have a direct effect on improving microbiota resilience. In this study, the microbiota of mice fed with a fiber enriched diet and challenged with antibiotic and *Clostridioides* (formerly *Clostridium*) *difficile* returned to pre-challenge composition whereas the microbiota of mice fed a low fiber diet and challenged in the same way did not (Hryckowian et al., 2018).

### Probiotics and Dietary Interventions Boosting Certain Microbial Species

Published data on human microbiota submitted to various challenges (Hsiao et al., 2014; Mardinoglu et al., 2018; Palleja et al., 2018) showed a number of species that are commonly perturbed. Those may be fragile species that could be boosted or replenished by nutritional intervention. For example, *Bifidobacterium adolescentis* is the most diminished species with high fat diet (Mardinoglu et al., 2018). In addition, this species is highly reduced and slow to recover following a challenge with antibiotics (Palleja et al., 2018). It is therefore reasonable to put forward and (i) test the hypothesis that providing *B. adolescentis* as component of a probiotic blend may improve the resilience of the microbiota and (ii) exclude the possibility that *B. adolescentis* is just a bioindicator. Other *Bifidobacterium* and *Lactobacillus* species that exist as probiotics such as *B. longum*, *B. bifidum*, *B. angulatum*, and *L. casei* are also altered by challenges to the microbiota (Mardinoglu et al., 2018; Palleja et al., 2018), although not as dramatically *as B. adolescentis*, and hence, could be part of a resilience blend nutritional solution. Not all challenge-diminished species exist as probiotics, but ingredients known to boost those species may also be used to prevent their decline during and after a challenge. For example, inulin favors the growth of *B. adolescentis*, and of *Faecalibacterium prausnitzii* (Ramirez-Farias et al., 2009). *F. prausnitzii* is a beneficial species that is diminished when the microbiota is challenged with antibiotics or high fat diet and that is associated with recovery from diarrhea (Hsiao et al., 2014; Mardinoglu et al., 2018; Palleja et al., 2018). Dietary interventions aimed at boosting certain species must be carefully dosed so that the targeted species does not become too prominent and negatively affect α-diversity, which will be against the desired effect.

Two studies investigated the effect of probiotics on perturbations of the microbiota by antibiotics to determine if they would improve resilience. One study showed no effect of supplementation with *Lactobacillus rhamnosus* and *Lactobacillus helveticus* on microbiota perturbations induced by the antibiotics amoxicillin + clavulanic acid (MacPherson et al., 2018) whereas a second older study showed that a mix of probiotics (*B. lactis* Bl-04, *B. lactis* Bi-07, *Lactobacillus acidophilus* NCFM, *Lactobacillus paracasei* Lpc-37, and *Bifidobacterium bifidum* Bb-02), minimizes the amoxicillin + clavulanic acid-induced disruption of fecal microbiota (Engelbrektson et al., 2009). The probiotic group in this study, showed a strong trend for improved recovery by random fragment length polymorphism (RFLP) analysis, and additionally a significant increase in resistance by bacterial culture.

Beyond repairing the microbiota composition, it is also relevant to consider how to minimize the effects of a perturbed microbiota on the host. Dysbiosis often leads to inflammation (Blander et al., 2017) and increased gut permeability (reviewed in Jandhyala et al., 2015), which in turn negatively impacts the microbiota. To break this vicious circle, interventions with a mix of probiotics containing species with proven anti-inflammatory properties such *as Lactobacillus rhamnosus* GG (Capurso, 2019) or shown to strengthen the gut barrier such as *Lactobacillus plantarum* (Karczewski et al., 2010) may be a good complementary approach to improving the microbiota by acting on the host physiology.

## Open Questions

Many questions remain to be answered on what other parameters make a microbiota resilient. The role of keystone species must be determined and may be important. These taxa are believed to interact with a large number of other taxa and may help maintain the status of the microbiota (Fisher and Mehta, 2014). At this point the role played by keystone species in microbiota resilience has not been clarified, and only a few candidate keystone species [e.g., *Ruminococcus bromii* (Ze et al., 2012)] have been proposed.

How about rare species? Typically, taxa with abundance below a certain threshold are rightfully excluded from analyses because the difference between a very low figure and zero might be due to method sensitivity and not to a physiological difference. This approach could be revisited for studies related to resilience, as the disappearance of low abundance taxa may be more relevant than a large decrease of an abundant one. If a low abundance species providing an important function becomes extinct, this irreversible event could shift the microbiota to a different stable state which may be a step toward dysbiosis.

The question remains open on whether knowledge at the species level is sufficiently reliable for intervention. The benefits of probiotics are known to be strain dependent. Therefore, providing a particular strain to replace a diminished species may not work, if it is another strain with a different function that is perturbed. Further improvements of the analysis techniques are needed since the current shotgun metagenomic microbiota analysis technology allows discrimination at the species level, but rarely at the strain level.

The role in microbiota resilience of gut microbes belonging to other kingdoms remains to be elucidated. Most studies of the microbiota have focused on bacteria, but archaea, eukaryotes, and viruses are also present in the gut. A few studies have found associations between the composition of virome, mycome or archaeome and human diseases, including type 2 diabetes, inflammatory bowel disease, and obesity (Blais Lecours et al., 2014; Bhute et al., 2017; Borges et al., 2018; Ghavami et al., 2018; Ma et al., 2018; Maya-Lucas et al., 2019; Zuo et al., 2019). Interestingly one study showed reduced virome diversity before development of autoimmunity in children susceptible to type 1 diabetes (Zhao et al., 2017). Very little is known about the interactions between kingdoms of the commensal gut microbes. To our knowledge there are no studies that suggest a role of viruses, archaea and fungi in mammalian gut microbiota resilience. Yet, it is possible that other organisms beyond bacteria play a role in resilience, especially phages since they have the ability to infect and lyse specific bacteria, thereby possibly controlling their numbers.

Another important question is whether the resilience of the gut microbiota is stress specific or is the degree of resilience similar regardless of the nature of the stress. At present this question has not been addressed experimentally.

## Closing Remarks

We summarized here the many challenges for understanding what makes a resilient microbiota and how to devise a strategy for an effective nutrition intervention to improve resilience. Improving microbiota resilience may have important beneficial effects on health. The current knowledge although incomplete, includes enough information to justify and guide the design of human trials of first-generation solutions. As we learn more about the mechanisms of microbiota resilience, future new findings will be used to design the composition of next-generation interventions.

## Author Contributions

All authors contributed to writing the manuscript, reviewed and approved its final version.

## Conflict of Interest

SD and SKD are full time employees of Société des Produits Nestlé. JD received consultancy honoraria from MaaT Pharma, LNC, Biofortis and Nestle and reports grants and/or lecture honoraria from Janssen, Sanofi, MaaT Pharma, BMS, Bridor and Danone.
